# Design and Optimization of a Hybrid-Driven Waist Rehabilitation Robot

**DOI:** 10.3390/s16122121

**Published:** 2016-12-14

**Authors:** Bin Zi, Guangcai Yin, Dan Zhang

**Affiliations:** 1 School of Mechanical Engineering, Hefei University of Technology, Hefei 230009, China; 18255187599@163.com; 2 Lassonde School of Engineering, York University, 4700 Keele Street, Toronto, ON M3J 1P3, Canada; dzhang99@yorku.ca

**Keywords:** waist rehabilitation robot, hybrid-driven, inverse kinematics and statics, optimization

## Abstract

In this paper a waist rehabilitation robot driven by cables and pneumatic artificial muscles (PAMs) has been conceptualized and designed. In the process of mechanism design, the human body structure, the waist movement characteristics, and the actuators’ driving characteristics are the main considerable factors to make the hybrid-driven waist rehabilitation robot (HWRR) cost-effective, safe, flexible, and well-adapted. A variety of sensors are chosen to measure the position and orientation of the recovery patient to ensure patient safety at the same time as the structure design. According to the structure specialty and function, the HWRR is divided into two independent parallel robots: the waist twist device and the lower limb traction device. Then these two devices are analyzed and evaluated, respectively. Considering the characters of the human body in the HWRR, the inverse kinematics and statics are studied when the waist and the lower limb are considered as a spring and link, respectively. Based on the inverse kinematics and statics, the effect of the contraction parameter of the PAM is considered in the optimization of the waist twist device, and the lower limb traction device is optimized using particle swarm optimization (PSO) to minimize the global conditioning number over the feasible workspace. As a result of the optimization, an optimal rehabilitation robot design is obtained and the condition number of the Jacobian matrix over the feasible workspace is also calculated.

## 1. Introduction

Nowadays, more and more people suffer the waist ache which is caused by human diseases, old age, or sedentary lifestyle. The great practical demand of rehabilitation physicians contributes to the development of the waist rehabilitation robot. For preventing secondary damage by the rigid links, soft actuators, like cable and pneumatic artificial muscle (PAM), are increasingly adopted [1].

The rehabilitation robot driven by cables was developed rapidly with a deep study of cable-driven parallel robots (CDDRs), and the CDDRs have favorable applicability because they feature high dynamics due to their small moving mass, large workspace, and low cost [2,3,4]. Surdilovic et al. presented STRING-man driven by seven cables for assisting locomotion recovery therapy and training [5]. However, there are some major drawbacks that exist, making the CDDRs often more difficult to design (e.g., it may be difficult to avoid cable interference) and more expensive to build and maintain when the number of cables and actuators is higher. On the other hand, the arrangement of cables should be more difficult in view of the recovery patient’s safety in this work [6,7]. Therefore, the number of cables can be reduced to a reasonable number.

Currently, PAM has been widely used in rehabilitation robots [8], since there are some advantages for PAM, such as a high force-to-weight ratio, variable installation possibilities, no mechanical parts, lower compressed air consumption, and low cost [9]. A parallel ankle rehabilitation robot driven by four PAMs was designed by Jamwal et al. [10], and since the application of PAM is limited by the contraction length and the unidirectional nature of force, the robot was optimized using a modified genetic algorithm [11].

For these reasons, and referring to [12,13], the human body structure, the waist movement characteristics, and the actuators’ drive characteristics are the main considerable factors in the process of mechanical structure design of the waist rehabilitation robot. It is well known that developing a rehabilitation robot suitable for task-oriented rehabilitation therapy is a major challenge encountered in the process of design [8]. Based on this, for the purpose of designing a cost-effective, safe, flexible, and well-adapted waist rehabilitation robot, a new mechanism for waist rehabilitation actuated by PAMs and cables is presented in this paper. The hybrid-driven waist rehabilitation robot (HWRR) is divided into two parallel robots according to the structure specialty and function: the waist twist device and the lower limb traction device, they drive the human body respectively and synergistically.

Considering that the human body has an effect on the structure of the rehabilitation robot, the waist and the lower limb can be considered as a spring and link, respectively [14]. Based on this, the inverse kinematics and statics of the HWRR are studied in this paper. Furthermore, since the performance of parallel robots greatly depends on their dimensions and the configuration of their actuators, the optimal design is analyzed separately based on the inverse kinematics and statics. In detail, in consideration of the contraction parameter of PAM, the optimal design of the waist twist device is presented. Ahead of the optimization problem formulation of the lower limb traction device, a brief discussion of the performance criteria and the constraints of the rehabilitation robot is presented. Then the optimization results are calculated using the condition number over the feasible workspace. Finally, we calculated the condition number of the Jacobian matrix over the feasible workspace and the cable length changes based on the designed trajectory.

The paper is organized as follows: Section 2 introduces the novel concept and details of the HWRR; Section 3 studies the inverse kinematic equations and static analysis of the waist twist device and the lower limb traction device; Section 4 shows the optimization of the HWRR; and, finally, conclusions and the proposed future works are discussed.

## 2. Structure Design

There seems to be a rather significant difference between rehabilitation robots and industrial robots in application and operation. The design objective of the HWRR is to make its features cost-effective, safe, flexible, and well-adapted. Thus, the human body structure and its complex motions were studied first [15]. The waist has three degrees of freedom (DOFs) relative to the pelvis, three directions of rotation, and the rotation of the Z axis direction is so small that it could be ignored. Thus, the motion between the waist and pelvis can be considered as two DOFs. Due to the flexibility of the human waist, it can be considered as a cylindrical helical spring. The freedoms of the waist can be considered equivalent to the axial load and biaxial bending of a spring. Similarly, the lower limb has three DOFs relative to the pelvic, three directions of rotation. Thus, the link imitates the lower limb, and the length of link varies with the individual.

As a matter of fact, the whole body motion forms a specific trace when people exercised their waist. Thus, for a better training effect, the waist and lower limb can be driven, respectively. It is considered that CDDRs could be adopted for traction of the lower limb due to the merit of a large workspace. As the limitation of the PAM applies in the rehabilitation robot, PAM is more suitable to drive the waist with the merit of high similarity to biological muscles. We name both the PAM and spring as the PAM system, which plays a vital supporting and securing role.

**Figure 1:**
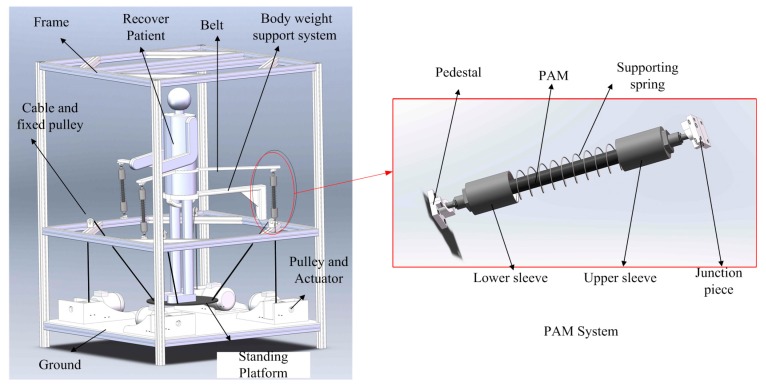
The structure of the HWRR.

The HWRR architecture introduced in this paper is shown in Figure 1. As mentioned above, the type of actuator, cable, and PAM are chosen first in the process of design, then we assume that the lower limb is driven by cables and PAM systems are used to drive the waist due to their respective driven characteristics. The waist twist device is composed of a belt and three PAM systems, and the lower limb traction device is composed of a standing platform and four cables. Some other components, including the frame, bodyweight support system, pulleys, and actuators, aim to make the HWRR safe, well-adapted, and flexible. The bodyweight support system fixed on the frame is used to hold the recovery patient’s pelvis stable. The PAM systems, the pulleys, and actuators are fixed to the frame.

The PAM system is composed of a pedestal, upper sleeve, lower sleeve, support spring, PAM, and junction piece. The pedestal is fixed to the frame through bolts, and the other end is connected to the lower sleeve with a spherical hinge. Similarly, the junction piece is fixed to the lower surface of the belt through bolts, and the other end is connected to the upper sleeve with a spherical hinge. The PAM is installed in the supporting spring, and the two ends of the spring connect with the upper sleeve and the lower sleeve, respectively. The upper sleeve and the lower sleeve are used to connect to other parts and guide the supporting spring and protect the PAM. As the belt connect with the top of the waist and it is driven by three PAM systems, the three PAM systems are designed to surround the recovery patient.

The recovery patient is required to stand on the standing platform, and then the height of the pelvis is adjusted through the four cable lengths, which are changed simultaneously. The lines connecting each conjunctive point between the cables and the standing platform forms a square. The other end of each cable passes round the pulley on the frame and twines on a pulley fixed to the ground. 

**Figure 2:**
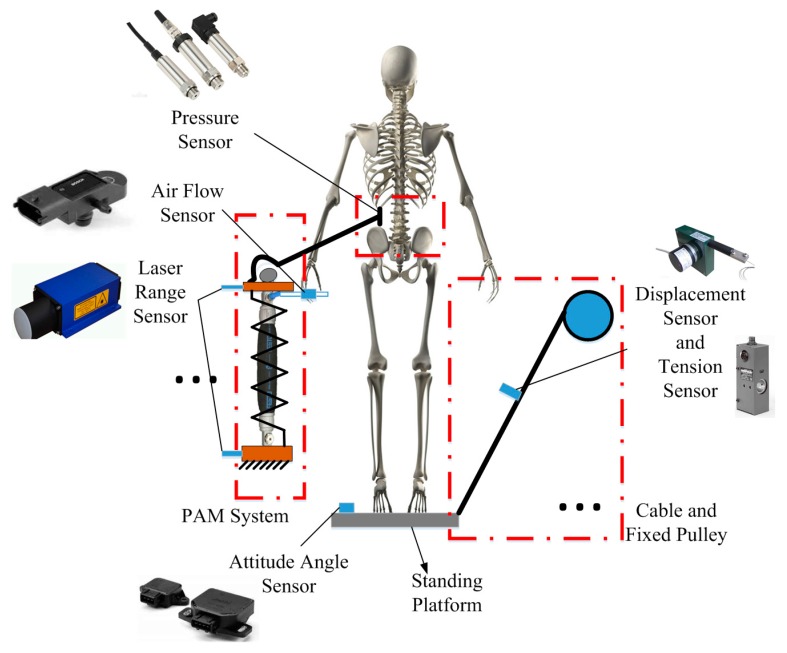
Assignment of the sensors.

It is important to ensure the safety and adaptability of the HWRR with respect to control. For more availability to different purposes and different recovery patients, there are some basic therapy strategies that should be applied by using the HWRR [16]. A variety of sensors are used to measure the position and orientation of the recovery patient to ensure patient safety. As shown in Figure 2, a pressure sensor is placed in the internal surface of the belt, a laser range sensor detects the change of the PAM system length, an air flow sensor is used to test the flow change of the PAM, the pose of the standing platform is detected by attitude angle sensors, the length change of the cable and the force acted on the cable are detected by displacement sensors and tension sensors, respectively.

**Figure 3:**
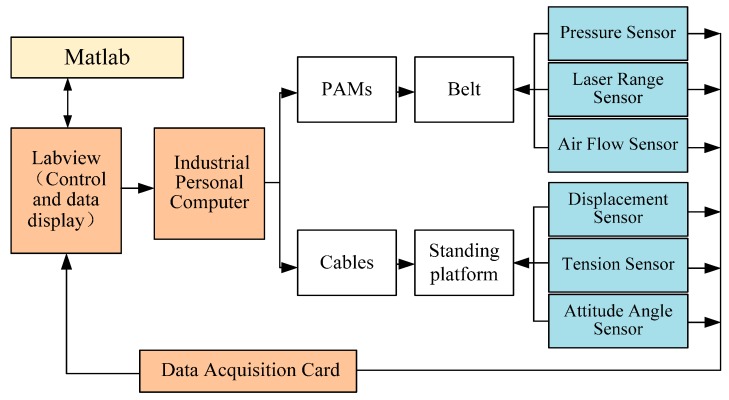
Control and measurement diagram of the HWRR.

Control and measure diagram of HWRR are designed and shown in Figure 3. The interactive interface is coded, the data of the cables and PAMs are sampled by a data acquisition card and processed by an industrial personal computer. 

## 3. Inverse Kinematics and Statics

Based on the analysis of the Section 2, the structure of the HWRR was divided into two parts: the waist twist device and the lower limb traction device. Inverse kinematics and statics of the HWRR can be carried out in the same way, respectively.

### 3.1. Inverse Kinematics and Statics of the Waist Twist Device

The function of the waist cannot be ignored when the waist twist device is working, and considering the structure of lumbar region and reacting force to the rehabilitation robot at the waist, the waist was considered as a spring in this paper. Thus, the structure of the waist twist device was similar as the parallel robotic neck proposed in [14]. However, by comparison, the change of the drive type brought different influences to the configuration, which is that the bottom of the PAM system and the lowest point of the waist are not in the same plane.

**Figure 4:**
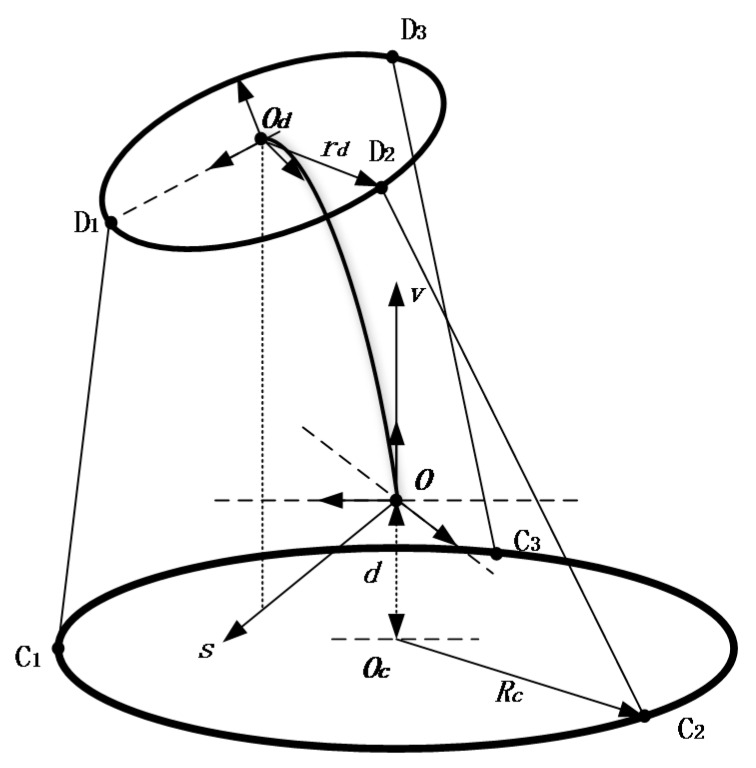
Coordinates and kinematic analysis of the waist twist device.

In order to describe the kinematic model of the waist twist device, the coordinate system is shown in Figure 4. The point of junction between the PAM system and the frame is $C_{i}\left(i=1,2,3\right)$, and the point of junction between the PAM system and the belt is $D_{i}$. A coordinate frame $O_{c}X_{c}Y_{c}Z_{c}$ is attached to the fixed base plane defined by the three bottom points of the PAM system, with the origin at the center of the three points. The y-axis is along $O_{c}C_{1}$, and the z-axis is perpendicular to the plane. We assumed that the bottom center of waist was at the right above of point $O_{c}$, and $\left|OO_{c}\right|=d$. Another coordinate frame OXYZ is attached to the plane that is parallel to the fixed base plane, with the origin at O and the y-axis is parallel with $O_{c}C_{1}$. A moving coordinate frame $O_{d}X_{d}Y_{d}Z_{d}$ is attached to the belt, with its origin at the top center of the waist. Since the points $C_{i}$ form a uniform distribution on a circle of radius $R_{c}$ centered at $O_{c}$, and $D_{i}$ forms a uniform distribution on a circle of radius $r_{d}$ centered at $O_{d}$, the coordinates of $C_{i}$ in the frame OXYZ are described as $C_{1}\left(0,R_{c},-d\right)$, $C_{2}\left(\frac{\sqrt{3}}{2}R_{c},-\frac{1}{2}R_{c},-d\right)$, $C_{3}\left(-\frac{\sqrt{3}}{2}R_{c},-\frac{1}{2}R_{c},-d\right)$, and the coordinates of $D_{i}$ in the frame $O_{d}X_{d}Y_{d}Z_{d}$ are described as $D_{1}\left(0,r_{d},0\right)$, $D_{2}\left(\frac{\sqrt{3}}{2}r_{d},-\frac{1}{2}r_{d},0\right)$, $D_{3}\left(-\frac{\sqrt{3}}{2}r_{d},-\frac{1}{2}r_{d},0\right)$. 

For the convenience of calculations, a planar body frame Osv is attached to the waist, with the origin at O, the s-axis is along $\vec{OO_{d}\prime }$, and the v-axis is the same as the Z-axis in the frame OXYZ.

The inverse position kinematics problem should be defined to calculate the PAM system lengths $H_{i}$ when the belt posture ${\left[\begin{array}{ccc} \varphi & \sigma & H_{b} \end{array}\right]}^{T}$ is given, where φ is the angle between axis s and axis X, σ is the angle between the horizontal plane and the belt, and $H_{b}$ is the vertical length of the bending waist. According to the analysis results of [14], we can easily obtain the inverse kinematics and statics of the waist twist device.

Since the belt posture is described by φ and σ, we can obtain the rotational matrix from the frame $O_{d}X_{d}Y_{d}Z_{d}$ to OXYZ when φ and σ are given and, consequently, the homogeneous transformation matrix from frame $O_{d}X_{d}Y_{d}Z_{d}$ to OXYZ can be obtained as:

(1) $$E=[\begin{array}{cccc} \sin^{2}\;\varphi +\cos\;\sigma \cos^{2}\;\varphi & (\cos\;\sigma -1)\cos\;\varphi \sin\;\varphi & \sin\;\sigma \cos\;\varphi & L_{s}\cos\;\sigma \\ (\cos\;\sigma -1)\cos\;\varphi \sin\;\varphi & \cos^{2}\;\varphi +\cos\;\sigma \sin^{2}\;\varphi & \sin\;\sigma \sin\;\varphi & L_{s}\sin\;\sigma \\ -\sin\;\sigma \cos\;\varphi & -\sin\;\sigma \sin\;\varphi & \cos\;\varphi & H_{b}\\ 0 & 0 & 0 & 1 \end{array}]$$

where $L_{s}$ is the s coordinate $O_{d}$ for in frame Osv, and it is a characteristic of the waist lateral bending, which is caused by the forces acting on the waist. Thus, $L_{s}$ should be solved first through a complicated quadratic equation proposed in [14], and the derivation process can be omitted in this paper.

By using the closed-vector-circle method, the PAM system lengths $H_{i}$ can be calculated with the formula:

(2) $$H_{i}=\|E\;\vec{O_{d}D_{i}}-\vec{OC_{i}}\|$$

where $\vec{O_{d}D_{i}}$ is the vector in the frame $O_{d}X_{d}Y_{d}Z_{d}$, and $\vec{OC_{i}}$ is the vector in the frame OXYZ.

**Figure 5:**
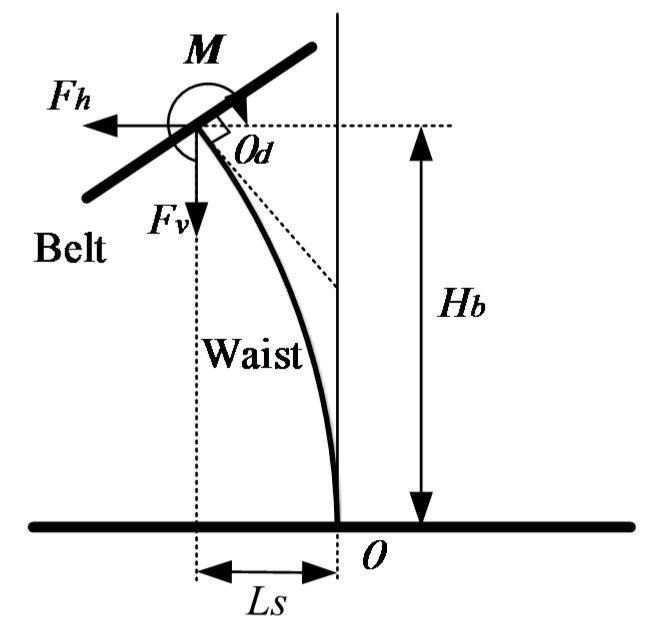
Force analysis of the waist.

As the waist plays an important role in the waist twist device, the waist can be considered in the static analysis, and the spring bending problem was first investigated by Timoshenko [16]. The forces acted on the belt can be simplified into two perpendicular forces $F_{h}$ and $F_{v}$ in the plane Osv, and a torque M perpendicular as shown in Figure 5. According to [14], the static formulation can be described as:

(3) $$W=X^{-1}B$$

where the items of matrix X are:

$x_{11}=L_{s}\cos\;\varphi +r_{d}e_{12}$,
$x_{12}=L_{s}\cos\;\varphi +\frac{\sqrt{3}}{2}R_{c}-\frac{\sqrt{3}}{2}r_{d}e_{12}-\frac{1}{2}r_{d}e_{12}$,
$x_{13}=L_{s}\cos\;\varphi -\frac{\sqrt{3}}{2}R_{c}+\frac{\sqrt{3}}{2}r_{d}e_{12}-\frac{1}{2}r_{d}e_{12}$,
$x_{21}=-R_{c}+L_{s}\sin\;\varphi +r_{d}e_{22}$,
$x_{22}=\frac{1}{2}R_{c}+L_{s}\sin\;\varphi -\frac{\sqrt{3}}{2}r_{d}e_{21}-\frac{1}{2}r_{d}e_{22}$,
$x_{23}=\frac{1}{2}R_{c}+L_{s}\sin\;\varphi +\frac{\sqrt{3}}{2}r_{d}e_{21}-\frac{1}{2}r_{d}e_{22}$,
$x_{31}=H_{b}+r_{d}e_{32}$,
$x_{32}=H_{b}-\frac{\sqrt{3}}{2}r_{d}e_{31}-\frac{1}{2}r_{d}e_{32}$,
$x_{33}=H_{b}+\frac{\sqrt{3}}{2}r_{d}e_{31}-\frac{1}{2}r_{d}e_{32}$.

***W*** and ***B*** are:

(4) $$W=\left[\begin{array}{c} t_{1}/H_{1}\\ t_{2}/H_{2}\\ t_{3}/H_{3} \end{array}\right],\;\;B=\left[\begin{array}{c} -\;F_{h}\cos\;\varphi \\ -\;F_{h}\sin\;\varphi \\ \;F_{v}-mg \end{array}\right]$$

Note that $e_{j,k}\left(j,k=1,2,3\right)$ is an item of the matrix E. $t_{i}$ is the force acting on the belt by the PAM system. The mass of the belt is taken as a mass point at the waist’s top center with quantity m, and the mass of the waist is ignored. Using Hooke’s law, the variable $F_{v}$ can be obtained:

(5) $$F_{v}=K\left(l_{0}-H_{b}\right)$$

where *K* is the waist equivalent stiffness, $l_{0}$ is the initial length of the equivalent spring of the waist.

### 3.2. Inverse Kinematics and Statics of the Lower Limb Traction Device

**Figure 6:**
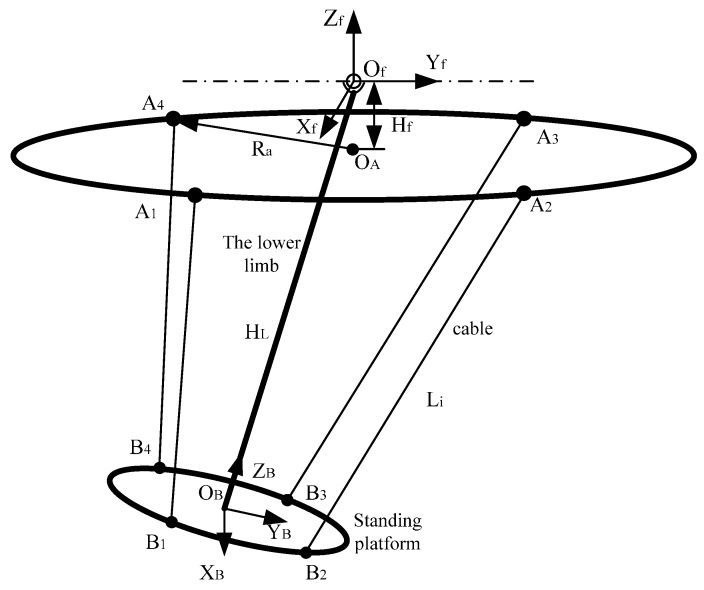
Coordinates and kinematic analysis of the lower limb traction device.

The lower limb traction device can be simplified to a cable-driven parallel robot shown in Figure 6. The lower limb of the recovery patient is considered as a link with three rotational degrees of freedom, and the rotation center at the top center of the link that is connected to the pelvis. We assumed that the link is perpendicular to the standing platform, and the four intersections of each cable with the frame are evenly distributed on a circle of radius $R_{a}$ centered at $O_{A}$; the four intersections of each cable with the standing platform are evenly distributed on a circle of radius $r_{b}$ centered at $O_{B}$. The rotational center of the link is right above $O_{A}$ with distance $H_{f}$. A coordinate frame $O_{f}X_{f}Y_{f}Z_{f}$ is attached to the pelvis, with the origin at $O_{f}$. A moving coordinate frame $O_{B}X_{B}Y_{B}Z_{B}$ is attached to the standing platform, with its origin at the center of the standing platform. 

Referring to Figure 4, the coordinates of $B_{i}$ in the frame $O_{B}X_{B}Y_{B}Z_{B}$ are described as $B_{1}\left(\frac{\sqrt{2}}{2}r_{b},-\frac{\sqrt{2}}{2}r_{b},0\right)$, $B_{2}\left(\frac{\sqrt{2}}{2}r_{b},\frac{\sqrt{2}}{2}r_{b},0\right)$, $B_{3}\left(-\frac{\sqrt{2}}{2}r_{b},\frac{\sqrt{2}}{2}r_{b},0\right)$, $B_{4}\left(-\frac{\sqrt{2}}{2}r_{b},-\frac{\sqrt{2}}{2}r_{b},0\right)$, and the coordinates of $A_{i}$ in the frame $O_{f}X_{f}Y_{f}Z_{f}$ are described as $A_{1}\left(\frac{\sqrt{2}}{2}R_{a},-\frac{\sqrt{2}}{2}R_{a},\;H_{f}\right)$, $A_{2}\left(\frac{\sqrt{2}}{2}R_{a},\frac{\sqrt{2}}{2}R_{a},\;H_{f}\right)$, $A_{3}\left(-\frac{\sqrt{2}}{2}R_{a},\frac{\sqrt{2}}{2}R_{a},\;H_{f}\right)$, $A_{4}\left(-\frac{\sqrt{2}}{2}R_{a},-\frac{\sqrt{2}}{2}R_{a},\;H_{f}\right)$.

Given the position $\left\{\begin{array}{ccc} \alpha & \beta & \gamma \end{array}\right\}$ of the standing platform, the inverse kinematics analysis allows us to calculate cable length $L_{i}\;\left(i=1,2,3,4\right)$. α, β, and γ are the angles of the standing platform between plane $O_{f}X_{f}Y_{f},\;O_{f}X_{f}Z_{f}$ and $O_{f}Y_{f}Z_{f}$, respectively. The cable length can be obtained as:

(6) $$L_{i}=\|\vec{A_{i}O_{f}}-\vec{B_{i}O_{B}}-\vec{O_{B}O_{f}}\|$$

where the vector $\vec{A_{i}O_{f}}$, $\vec{B_{i}O_{B}}$ and $\vec{O_{B}O_{f}}$ are described in the frame $O_{f}X_{f}Y_{f}Z_{f}$.

According to the force balance condition of standing platform, the equations should be satisfied:

(7) $$\{\begin{array}{c} F_{s}+\sum_{i=1}^{4}F_{i}=0\\ \sum_{i=1}^{4}\left({}^{o}m_{i}\times F_{i}\right)+\sum M_{ext}=0 \end{array}$$

where ${}^{o}m_{i}$ is the arm of cable tension $F_{i}$, $M_{ext}$ is the resultant torque acting on the standing platform by the lower limb, and described as $M_{ext}={\left[\begin{array}{ccc} M_{x0} & M_{y0} & M_{z0} \end{array}\right]}^{T}$. Let $F={\left[\begin{array}{cc} \begin{array}{cc} F_{1} & F_{2} \end{array} & \begin{array}{cc} F_{3} & F_{4} \end{array} \end{array}\right]}^{T}$ represent the cable tension vector.

Assuming that no more force and torque acted on the standing platform, the relationship between F and $M_{ext}$ is:

(8) $$M_{ext}=J_{B}^{T}F$$

where $J_{B}$ is the Jacbian matrix of the cable-driven parallel robot and can be described as:

(9) $$\{\begin{array}{c} J_{B}={\left[\begin{array}{cc} \begin{array}{cc} J_{B1} & J_{B2} \end{array} & \begin{array}{cc} J_{B3} & J_{B4} \end{array} \end{array}\right]}^{T}\\ J_{Bi}=\vec{B_{i}O_{B}}\;\frac{L_{i}}{l_{i}}\left(i=1,2,3,4\right) \end{array}$$

As $J_{B}^{T}$ is not a square matrix, the generalized inverse matrix can be used to calculate F and Equation (8) can be expressed as

(10) $$F={\left(J_{B}^{T}\right)}^{+}M_{ext}=\bar{J}M_{ext}$$

where $\bar{J}={\left(J_{B}^{T}\right)}^{+}=J_{B}{\left(J_{B}^{T}J_{B}\right)}^{-}$.

## 4. Optimization

Developing an optimization scheme to obtain an optimum arrangement of the actuator attachment points on the frame is the main objective of this section. This means that the goal is to obtain an optimal robot design that satisfies all of the defined constraints for waist rehabilitation. This section has been divided into two parts based on the different functional structure.

### 4.1. Optimization of the Waist Twist Device

Gao et al. [14] proposed a cable-driven parallel robot with a spring spine, and the optimal design and workspace of the robot are analyzed with the positive cable tension constraint. In detail, the parallel mechanism was optimized in order to minimize the actuation force to decrease the size of actuators and reduce energy consumption. The optimization variables are $r_{d}$ and $R_{c}$, which represent the cables’ end positions at the fixed base and moving platform. The optimal results show that it is better to place all of the cables near the upper bound to obtain a minimum actuation force corresponding to either energy consumption or actuation size, and the best solution has a radius ratio $r_{d}/R_{c}$ close to one. 

According to the existing research on PAMs [8,9], we know that the limitation of the change of PAM length should be taken into account, and the change of the PAM length is quite limited for preventing damage, itself. The maximum allowable contraction length is 25% of the original length. Since the workspace required is symmetrical, this determined that the original length $H_{ori}$ of the PAM is 90% of the maximum length $H_{max}$.

Here the required workspace is defined as the value of σ changed between 0° and 20°. We found that the value of σ reflects the change of PAM length $H_{i}$. We assume that the waist twist device is in the initial position when σ=0°, and in this context the shrinkage of the PAM’s greatest length can be calculated with σ=20°. 

On the basis of existing research results, and the inverse kinematic and statics, the waist twist device was optimized. First of all, we should calculate the force of the PAM system when the device is in the position ${\left[\begin{array}{ccc} 90{}^{\circ} & 20{}^{\circ} & 0.13\;m \end{array}\right]}^{T}$. Let us assume that the parameters of the equivalent spring of the waist are shown in the Table 1. Therefore, the spring constant K=5882 N/m and the moment of inertia I of the cross-section of the spring wire and the flexural rigidity $\alpha_{0}$ can be calculated according to [17]. The results calculated were $6.135\times 10^{-11}\;m^{4}$ and 0.1419, respectively. Other necessary parameters are chosen as $R_{c}=r_{d}=\;0.75\;m$ and the mass of the moving platform is m=1 kg. Based on the results above, the numerical results of $L_{s}$, t1′=t1H1 and d can be calculated as 0.0262 m, 2415.6 N/m, and −0.0264 m. This means that the original length of PAM is 0.154 m. 

Based on the above qualitative analysis, the type we chose is a Festo Fluidic Muscle DMSP-10-154N-RM-RM. Properties of this muscle are as follows: the inside diameter is 10 mm and nominal length is 154 mm. PAM has a radial pneumatic connection, pressed end caps, and integrated air connectors. The maximum working pulling forces is 630 N.

### 4.2. Optimization of The Lower Limb Traction Device 

Ahead of the optimization problem formulation of the lower limb traction device, a brief discussion on the robot performance criteria and their significance is given, which leads to the formation of the present optimization problem. In the following pages, three important criteria are discussed. 

(1) Workspace

For cable-driven parallel robots, it is not only necessary to solve the closure equations but it is also essential to verify that the equilibrium can be achieved with non-negative cable forces. Thus, the workspace W of the lower limb traction device is defined as the subset of space where the tensions on cables are both non-negative. Based on the static analysis, the factors affecting the workspace are the length of lower limb and the cable attachment points. Expanding further, the workspace can be described by the following formula [18]:

(11) $$W=\left\{\alpha ,\beta ,\gamma \in ℛ|F_{i}>0\right\}\left(i=1,2,3,4\right)$$

The workspace is not only the constraint condition, but related to the criteria in the following sub-sections. Ahead of the calculation, the feasible workspace should be defined first. We assumed that the feasible workspace created by rotating the standing platform along x-, y-, and z-axes through incremental rotations within the ranges of ±20°, ±20°, and 0°, respectively. 

(2) Condition Number

It is well known that the Jacobian matrix $J_{B}$ maps the joint velocities of the robot to its Cartesian velocities. The condition number k ($J_{B}$) of Jacobian matrix is a local measure of its sensitivity to the changes in the kinematic variables of the robot and depends only on the robot’s physical configuration.

To evaluate the robot design, the condition number is generally obtained at each individual point in the feasible workspace region, for a given orientation of the end effector. The Jacobian matrix was found to be a 4 × 3 non-square matrix due to the redundant actuation. Thus, the singular values of the Jacobian matrix should be calculated as:

(12) $$\mu_{i}=\sqrt{\lambda_{i}\left(J_{B}^{T}J_{B}\right)}\left(i=1,2,3\right)$$

where $\lambda_{i}$ is the singular values of $J_{B}^{T}J_{B}$, and dictated that $\mu_{1}\geq \mu_{2}\geq \mu_{3}\geq 0$.

Thus, the condition number of the Jacobian matrix is described as:

(13) $$1\leq k\left(J_{B}\right)=\frac{\mu_{1}}{\mu_{3}}<\infty$$

It is easily found that the smallest possible value of the condition number is 1, and poses with a condition number of 1 are called isotropic poses. On the contrary, the condition number is said to be ill conditioned.

Instead of considering the $k\left(J_{B}\right)$ in a specific pose, a global condition number (G) over the manipulator workspace W is normally used [19] and expressed as:

(14) $$G=\frac{\int_{W}k\left(J_{B}\right)dw}{\int_{W}dw}$$

*G* is bounded by the range of $k\left(J_{B}\right)$. The index *G* represents the average behavior of the condition number over the feasible workspace W, and the maximum value of the condition number in the entire workspace can be obtained and minimized.

(3) Cable Force

The cable force can be minimized in order to decrease the size of the actuators and reduce energy consumption. Based on [14], there are two objective functions and measures to optimize the cable force. The two objective functions we called minimum average *F* and min-max ***F***. The two measures can be expressed as:

(15) $$F_{ma}=\sqrt{\overset{4}{\underset{i=1}{\sum }}{F_{i}}^{2}}$$

(16) $$F_{mm}=max\left\{F_{1},F_{2},F_{3},F_{4}\right\}$$

In this paper, to minimize energy consumption, we prepare to minimize the average *F* over the feasible workspace using Equation (15). Thus, the minimum average *F* objective function is defined as:

(17) $$U=\frac{1}{W}\int_{W}F_{ma}dW$$

With the minimum peak value of the condition number in the entire workspace ***W***, the final *U* represents the average behavior of the average cable forces F over the feasible workspace.

However, note that the limits of the cable forces can be determined using the singular value decomposition theorem and are given as:

(18) $$\frac{\left\|M_{ext}\right\|}{\mu_{1}}\leq \|F_{i}\|\leq \frac{\left\|M_{ext}\right\|}{\mu_{3}}$$

This shows that the maximum of the cable forces is governed by the minimum singular value of $\mu_{3}$ and the cable forces can be reduced by maximizing this value. However, the minimum singular value automatically gets maximized when the maximum condition number is minimized [11]. Thus, the measured min-max F can be ignored.

As to the analysis above, and based on the inverse kinematics and statics in Section 3.2, the optimization variables come to be $R_{A}$, $r_{B}$, and $H_{f}$, which represent the cables’ end positions at the fixed frame and standing platform, and the distance between points $O_{f}$ and $O_{A}$. Additionally, all of the variables should be specified (α, β, and γ), and without loss of generality, the variable $H_{L}$ is assumed as 0.085 m  because different $H_{L}$ can be obtained by pretightening all four cables simultaneously. Thus, the optimization of the lower limb traction device is a constrained optimization problem. It is generally stated as:

(19) $$\begin{array}{c} \mathrm{Minimize}\;\phi \left(r_{B},R_{A},H_{L}\right)=G\\ \mathrm{subject}\mathrm{to}\begin{array}{c} r_{l}\leq r_{B}\leq r_{u}\\ R_{l}\leq R_{A}\leq R_{u}\\ h_{l}\leq H_{L}\leq h_{u} \end{array},\;\mathrm{and}F_{i}\geq 0\left(i=1,2,3,4\right) \end{array}$$

Note that $r_{l}$, $R_{l}$, $h_{l}$, $r_{u}$, $R_{u}$, and $h_{u}$ are the lower and upper bounds for $r_{B}$, $R_{A}$, and $H_{f}$, respectively. These values are based on the size of the human trunk, and in the numerical calculation they are selected as $r_{l}=0.2\;m$, $R_{l}=0.6\;m$, $r_{u}=0.36\;m$, and $R_{u}=0.8\;m$. For preventing interference between the lower traction device and the waist twist device, the remaining values are selected as $h_{l}=-0.3\;m$ and $h_{u}=0\;m$. Let the length of the lower limb be 1 m and, for convenience, the feasible workspace and G are discretely defined in the process of optimization.

Particle swarm optimization (PSO) is a computational method that optimizes a problem by iteratively trying to improve a candidate solution. The quality of the solution is evaluated by fitness. It can be used to solve multi-objective problems, and can reach a similar solution in less time [20].

As mentioned before, it is suitable to apply PSO because of the derivation of the objective function and the constraint conditions are difficult.

First of all, we transformed constrained optimization problems to unconstrained optimization problem by using penalty function method firstly, and then we obtained the optimal resolution based on PSO.

**Figure 7:**
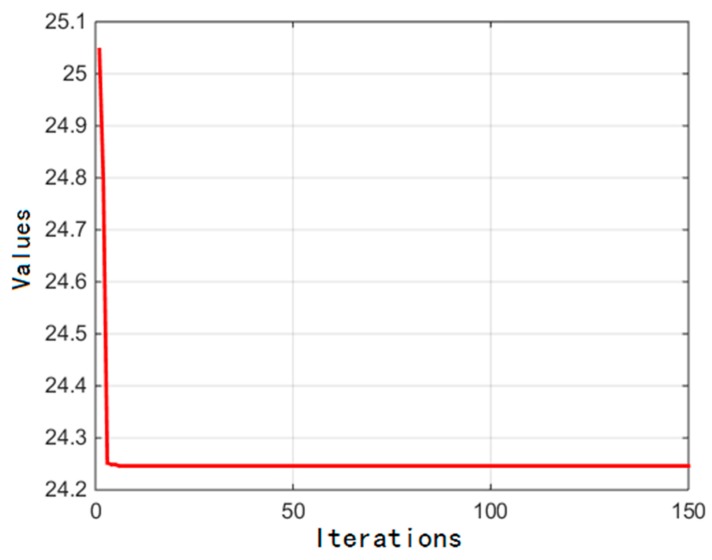
The value change of the objective function.

The numerical optimization results are 0.2 m, 0.7187 m, and −0.3 m. The minimum of $\phi \left(r_{B},R_{A},H_{L}\right)$ is 24.2460. The value changes of the objective function are shown in Figure 7. It is shown that the convergence of PSO is good. The value changes of the objective function degraded greatly when the iterations were smaller than 10, and changed to be steady in the least time. The results demonstrate that PSO used in this paper has better robustness and adaptability.

**Figure 8:**
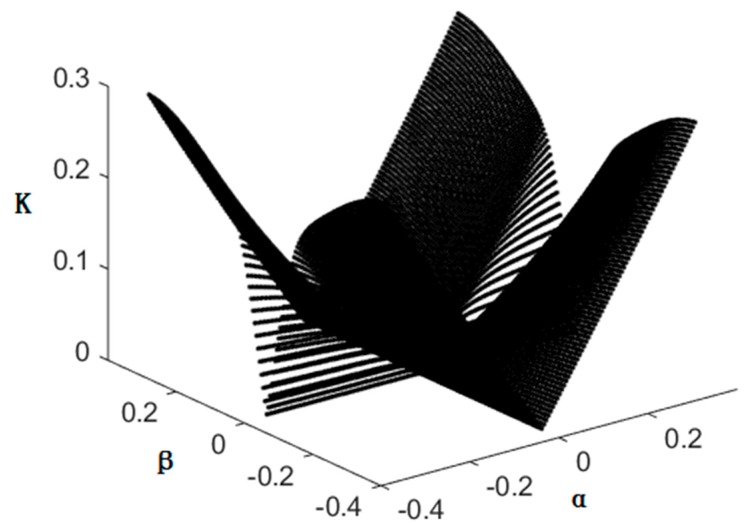
Condition number at γ=0°

The condition number of the Jacobian matrix over the feasible workspace are calculated with the optimization results and display the reciprocal value of $k\left(J_{B}\right)$ in Figure 8, which can show the characteristic visually. We can see that the values of $k\left(J_{B}\right)$ approach 0 when α or β are equal to 0, that is to say the lower limb traction device is in a singular configuration, and in other positions the condition numbers of the Jacobian matrix are far from 0; in other words, the lower limb traction device is said to be well-conditioned after the optimization. 

**Figure 9:**
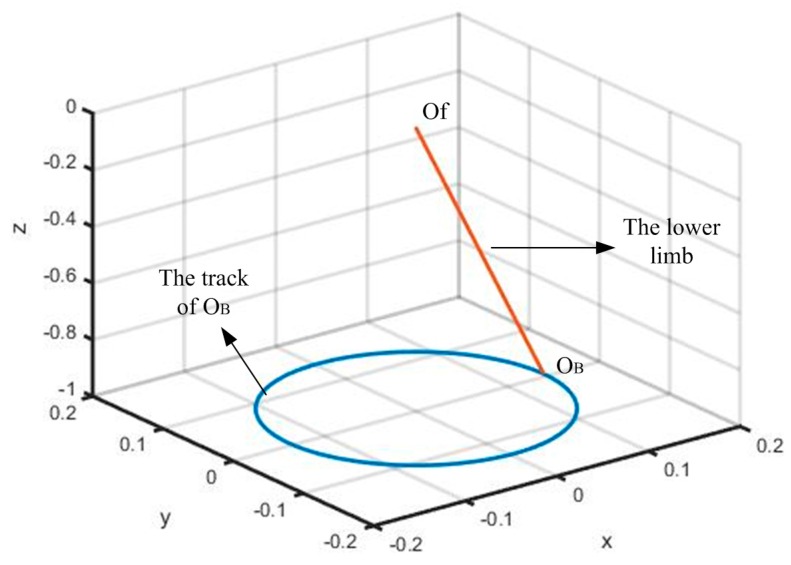
The track of the standing platform.

Based on the optimization results, the cables’ lengths are calculated when the track of the standing platform is designed beforehand and the track of the standing platform is designed based on the rehabilitation strategy. We assumed that the center of gravity of the pelvis is a circle when a healthy person exercises their waist. Since the pelvis is fixed by the bodyweight support system in the aforementioned design, the track of the standing platform is set to be a circle to simulate the pelvis’ circular movement during the rehabilitation training (see Figure 9). Note that the angle between the lower limb and the vertical line is set to be 8°. 

**Figure 10:**
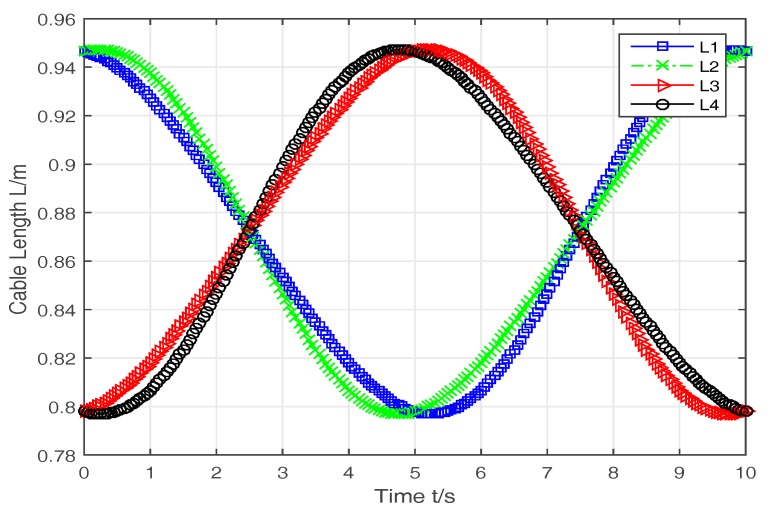
The change of the cables’ lengths.

The cables’ lengths are calculated based on the results of the kinematic analysis and the track of $O_{B}$. The results are shown in Figure 10. It is shown that the change curves of the cable’s lengths are continuous and smooth, and it meets the design requirements of the rehabilitation robot.

## 5. Conclusions

We presented a new mechanism driven by cables and PAMs for waist rehabilitation. Considering the waist kinematic features and characters of the actuators, the waist twist device and the lower limb traction device are adopted to make the HWRR cost-effective, safe, flexible, and well-adapted. A variety of sensors are chosen and their locations are designed to measure the position and orientation of the recovery patient to ensure their safety at the same time as the structure design. The coordination control of these two device should be encouraged to validate the effect of the HWRR. The waist and the lower limb are considered as a spring and link, respectively. The inverse kinematic and statics of the waist twist device and the lower limb traction device are analyzed for the subsequent optimization. The PAM system’s position on the frame is obtained by the optimization of the waist twist device with the contraction parameter of PAM being considered. The lower limb traction device is simplified to a CDDR driven by four cables. Ahead of the optimization of the lower limb traction device, the workspace, condition number of Jacobian matrix, and the cable forces are discussed and their significance is given in Section 4.2, which leads to the formation of the present optimization problem. As the maximum of the cable forces is governed by the minimum singular value of $\mu_{3}$, and the minimum singular value can be automatically maximized when the maximum condition number is minimized, the condition number of Jacobian matrix over the feasible workspace is selected to be optimized. Then the optimization problem is described as a formula which is solved by PSO. Optimization results show that PSO has good robustness and adaptability to solve the constrained problems. Finally, we calculated the condition number of the Jacobian matrix over the feasible workspace and the cable lengths change based on the designed trajectory. The lower limb traction is shown in the singular when α or β are equal to 0, and the condition number of the Jacobian matrix is larger than 0.1 in other positions, that is to say, the lower limb traction device is said to be well-conditioned when α or β are not equal to 0. The cable length change curves show that the change of the cable lengths are smooth and continuous.

For future work, we will study the waist structure and kinematics by experimentation for improvement of the design of the HWRR. Based on this, the trajectory plan based on the dynamics and coordinated control method of cables and PAMs will be analyzed in future work, and for producing a reliable and multifunction rehabilitation robot for clients, the experimental verification will be done through a large number of experiments.

## Figures and Tables

**Table 1:** The parameters of the equivalent spring of the waist.

The Initial Length $l_{0}$	0.18 m
the mean diameter $D_{m}$	0.1 m
the pitch s	0.02 m
the shearing modulus G	80,000 MPa
the elastic modulus E	20,500 MPa
the diameter of the spring wire d	0.005 m

## Nomenclature

$C_{i}$	point of junction between PAM system and frame
$D_{i}$	point of junction between PAM system and belt
$R_{c}$	radius of the circle center round $O_{C}$
$r_{d}$	radius of the circle center round $O_{D}$
d	distance between point O and point $O_{c}$
φ	angle between axis s and axis X
σ	angle between the horizontal plane and the belt
$H_{b}$	vertical length of the bending waist
$L_{s}$	the s coordinate $O_{d}$ for in frame Osv
$H_{i}$	PAM system length
$F_{h}$	perpendicular force acted on the belt
$F_{v}$	force acted on the belt in the plane Osv
M	torque acted on the belt
K	the waist equivalent stiffness
$l_{0}$	the initial length of the equivalent spring of the waist
$A_{i}$	point of junction between cable and frame
$B_{i}$	point of junction between cable and the standing platform
$R_{a}$	radius of the circle center round $O_{A}$
$r_{b}$	radius of the circle center round $O_{B}$
$L_{i}$	cable length
*α*	angle between the standing platform and plane $O_{f}X_{f}Y_{f}$
*β*	angle between the standing platform and plane $O_{f}X_{f}Z_{f}$
*γ*	angle between the standing platform and plane $O_{f}Y_{f}Z_{f}$
$F_{i}$	cable tension
$M_{ext}$	the resultant torque acted on the standing platform by the lower limb
$J_{B}$	Jacbian matrix of the cable-driven parallel robot
W	workspace
$\mu_{i}$	the singular values of the Jacobian matrix
